# Feasibility of Establishing Community Living Labs (CoLLabs) to Improve Access to Prostate Health Resources, Education, Amenities, and Community Health: Protocol for a Pragmatic Clinical Trial

**DOI:** 10.2196/89330

**Published:** 2026-05-15

**Authors:** Folakemi T Odedina, Opeyemi Bolajoko, Quincy Wimberly, Daniel Lee, Floyd Willis, Toshiko Moutlrie, Wayne Ford, Derry Green, Arnold Merriweather, Jocelyn Turner, Michelle Fudge

**Affiliations:** 1 Hematology/Oncology Department, Mayo Clinic in Florida, Jacksonville, FL, United States; 2 Cancer Health Equity Research Program, Mayo Clinic in Florida, Mayo Clinic Community Health Collaborative, 214 N. Hogan St. Suite 170, Jacksonville, FL, 32202, United States, 1 9046669242; 3 Quantitative Health Sciences, Mayo Clinic in Florida, Jacksonville, FL, United States; 4 Family Medicine, Mayo Clinic in Florida, Jacksonville, FL, United States; 5 American Legion Post 197, Jacksonville, FL, United States; 6 American Legion Post 244, Jacksonville, FL, United States; 7 American Legion Post 194, Saint Augustine, FL, United States; 8 Turner Alliance Consulting, Jacksonville, FL, United States

**Keywords:** prostate cancer, community living labs, learning health system, black men, community health workers, social determinants of health

## Abstract

**Background:**

In 2022, nearly 60,000 prostate cancer (CaP) cases were reported among Black men, who face an estimated lifetime risk of approximately 1 in 6, compared with 1 in 8 among White men. The disproportionate burden of CaP in Black men has been attributed to a combination of health-system factors, variations in care processes, and individual- or patient-level determinants. Addressing these multilevel contributors will require innovative strategies to advance prostate health equity.

**Objective:**

In this paper, we discuss the protocol for assessing the feasibility of establishing a Community Living Lab (CoLLab) Learning Health System in Black communities and the impact on facilitating access to prostate health Resources, Education, Amenities, and Community Health (REACH) services for Black men.

**Methods:**

The proposed research design for the study is a pragmatic clinical trial. The research setting is Northeast Florida, with the intervention based in 3 American Legion Posts (ALPs) and the control in one ALP. The development of the CoLLab REACH intervention was guided by the Intervention Mapping Framework, focusing on program design, adoption, implementation, and monitoring and evaluation plan. The intervention was cocreated with community members and is being provided by community health workers. The primary outcomes are improvement of Black men’s CaP awareness, knowledge, attitude, health beliefs, perceived control, intentions, cues to action, and clinical trials’ awareness. Generalized linear mixed regression approaches will be used to determine the differences between the study variables for the intervention and control groups.

**Results:**

The CoLLab study was awarded in September 2023 and received institutional ethics committee approval on March 20, 2024. CoLLab REACH intervention includes the following services: (1) Wellness RX program, implemented to address financial health needs and grocery distribution to address food deserts of the communities around the posts; (2) social determinants of health Navigation Services that include food, housing, transportation, employment aid, legal aid, and financial support based on zip code; (3) educational resources and videos on CaP prevention, screening, detection, treatment, and survivorship; (4) The Clinical Trials Matching Services to match participants to Mayo Clinic clinical trials and biomedical research; and (5) CaP Advocacy training program. Baseline recruitment for the intervention arm was completed in June 2025, with 183 Black men enrolled at ALPs. By June 2025, 11 participants were enrolled at the control ALP, and recruitment for the control arm remains ongoing. Data analysis will be conducted upon completion of follow-up assessments at months 4, 8, and 12.

**Conclusions:**

We successfully co-designed the CoLLab REACH services at 3 ALPs. Using a longitudinal pretest-posttest design, we will assess the intervention’s impact at both the individual and community levels to evaluate the feasibility of this community-based, culturally informed approach. In addition, we will examine the feasibility of replicating the CoLLab Learning Health System in underserved communities nationwide.

## Introduction

The disproportionate burden of cancer that exists in different populations is due to multiple factors, including clinical, cultural, behavioral, psychosocial, biological, environmental, and socioeconomic factors [1]. These cancer burdens are often magnified in special populations (communities of color, persons with a migration background, communities of Latin heritage), geographical areas (rural communities), and other marginalized or underserved communities. According to the American Cancer Society, inequalities in cancer care and outcomes have persisted for people of African descent populations for decades, including African Americans, foreign-born Blacks, and Afro Latinos [2]. Cancer is the second leading cause of death for people of African descent in the United States, with prostate cancer (CaP) leading in estimated new cancer cases and second in estimated new cancer deaths among Black men [2]. In 2022, nearly 60,000 CaP cases were reported among Black men, who face an estimated lifetime risk of approximately 1 in 6, compared with 1 in 8 among White men (WM). Several reasons for the significant burden experienced by Black men have been documented in the literature and well summarized by the Institute of Medicine’s 2003 report on Unequal Treatment [3], including (1) health-systems factors such as quality of care coordination, cultural competency of staff, and coordination of care; (2) care process factors, such as actual/perceived bias, level of involvement of the patient in decisions, interaction with the patient, knowledge, and time pressure; and (3) individual/personal level factors such as cultural factors, psychosocial factors, decision-making/preferences, comprehension, past experience, and perceived behavioral control in accessing the system. The latter underscores the role of social determinants of health (SDOH) in health disparities and inequities. One of the recommendations from the Institute of Medicine report is the use of community health workers (CHWs*)* as community navigators for medically underserved and racial and ethnic minority populations. However, there is limited access to CHWs with specialization in CaP education and navigation in minoritized and marginalized communities.

There are three primary challenges of existing CaP interventions targeting Black men: (1) limited comprehensiveness of interventions in addressing CaP care and survivorship (CaPCaS) and clinical trials diversity; (2) use of locations or channels that are not easily accessible by Black men; and (3) lack of maintenance and sustainability beyond the study intervention period. To address these challenges, we proposed creating Community Living Lab (CoLLab) Learning Health Systems in Black communities. Pallot et al [4] defined Living Lab as an *“*innovation platform that brings together all stakeholders at the earlier stage of the innovation process in order to experiment breakthrough concepts and potential value for both the society and users that will lead to breakthrough innovations.” According to the European Network of Living Labs, it is a system and environment for building a future economy in which real-life user-centric innovation will be the normal cocreation technique for new products, services, and societal infrastructures. The key concepts of a Living Lab are systems thinking, functional environment, real world, innovation, collaboration, engagement, inclusive research, actionable research, and sustainability. In an integrative review of Living Labs for health, Junghee et al [5] found that the approach improved quality of life, physical and social health, and cognitive function of the populations studied. It is worth noting that the majority of the Living Labs were in Europe. Despite the potential of the Living Lab approach in fostering sustainable interventions in minoritized and marginalized communities, it is an untapped and untested health care innovation in addressing cancer disparities in the United States. We view testing the Living Lab approach as an innovative strategy for addressing CaP, with strong potential to establish a community-based system that delivers comprehensive prostate health Resources, Education, Amenities, and Community Health (REACH) services.

The CoLLab intervention will serve as a Learning Health System in urban Black communities. According to the Agency for Healthcare Research and Quality [6], a learning health system is “a health system in which internal data and experience are systematically integrated with external evidence, and that knowledge is put into practice.” The characteristics include (1) leaders committed to a culture of continuous learning and improvement; (2) use of evidence in real-time to guide care; (3) use of IT to share new evidence that will improve decision-making; (4) inclusion of vital members of the learning team; (5) use of data and care experiences to improve care; and (6) continuous assessment of outcomes to refine processes and training. This is an iterative journey that includes a feedback cycle for learning and improvement. The CoLLab will provide an opportunity to innovatively create Learning Health Systems in Black communities.

To be successful, the CoLLab Learning Health System relies on CHWs and navigators. CHWs and navigators are well-trained professionals who form the bridge between communities and clinicians. Black men who work with CHWs and navigators typically have better access to health services, gain knowledge about cancer, cancer prevention, and treatment, and see better overall health outcomes [7-9]. Training trusted community members to discuss health matters is another community-based method. Barbers, faith leaders, and cancer survivors in the community have been trained as citizen scientists to understand cancer screening and treatment and develop ways to speak on health subjects in an approachable way that is both engaging and culturally informed [10-14]. The trained CHWs will be crucial to the success of the study.

The CoLLab Learning Health System will also address the digital health divide in this era of decentralized services and telehealth. Internet-based telehealth programs, mobile apps, artificially intelligent avatars, artificial/virtual reality, health kiosks, and web-based decision aids are common tools used in digital health. The COVID-19 pandemic required clinicians and health care providers to adapt to the unique circumstances of social distancing and adopt more of these tools. Although digital health technologies were more prevalent, they did not reduce health disparities [15]. Barriers to access remained prevalent in this new landscape. While Black men own and use desktop and laptop computers and smartphones equally or more than non-Hispanic WM, their access to the internet, particularly in rural regions, is less than that of non-Hispanic WM [15-17]. Other sociodemographic factors that negatively drive the digital health divide include: age-related factors such as inexperience and unfamiliarity with technology or low vision causing difficulty to read text on small screens; the inability to afford internet or pieces of technology; or face literacy challenges due to fewer years of formal education or English not being their primary language [16]. Cultural perceptions about technology also affect Black men’s use of digital health tools. For some Black men, the internet is not seen as a means of health communication. Speaking or responding to an avatar might feel unnatural and may be perceived as not credible as interactions with physicians [16,18]. The technology itself may be designed or programmed in a way that Black men may not find engaging [16]. Highly clinical language, unfriendly user design and presentation, inaccurate or outdated information create unintentional barriers, while highly interactive, user-driven, digital resources with culturally relevant images and icons lessen the divide [16,19]. The CoLLab Learning Health System will provide an opportunity to introduce Black men to digital health, addressing the barriers that we have highlighted.

A notable aspect of the CoLLab study is its use of a precision community health framework. Precision community health involves tailoring health interventions to specific communities, considering social, cultural, behavioral, and environmental contexts [19-21]. The emphasis is on community-level engagement and implementation, rather than just population-level analytics. The key characteristics include (1) use of community-specific data; (2) focus on engaging the community in designing and delivering interventions; (3) interventions that are context-sensitive and community-tailored, not just statistically targeted; and (4) involvement of community-based participatory research.

The primary objective of this study is to assess the feasibility of establishing a CoLLab Learning Health System within 3 American Legion Posts (ALPs) as *“*community connector sites” that can facilitate access to prostate health REACH services for Black men. The American Legion is the nation’s largest wartime veterans service organization aimed at advocating patriotism across the United States through diverse programs. With a mission to enhance the well-being of America’s veterans, their families, military, and communities through devotion to mutual helpfulness, the American Legion has more than 12,000 Posts in communities throughout the United States and over 200 posts in Florida. The specific aims of the study are (1) to cocreate real-life user-centric CoLLab Learning Health System at 3 ALPs; (2) using a pretest-posttest design, evaluate the impact of the System at the individual and community levels; and (3) to explore the feasibility of replicating the CoLLab Learning Health System in Black communities nationally.

## Methods

### Study Design

#### CaPCaS Models

**Figure 1.**
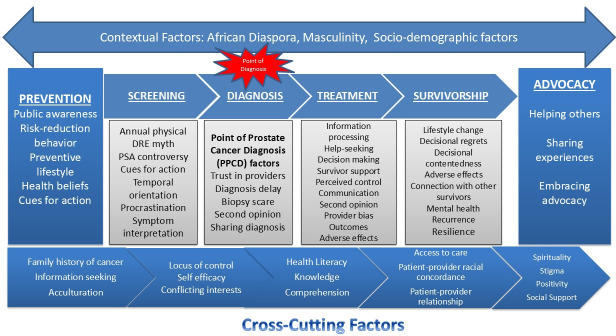
Prostate cancer care and survivorship model.

**Figure 2.**
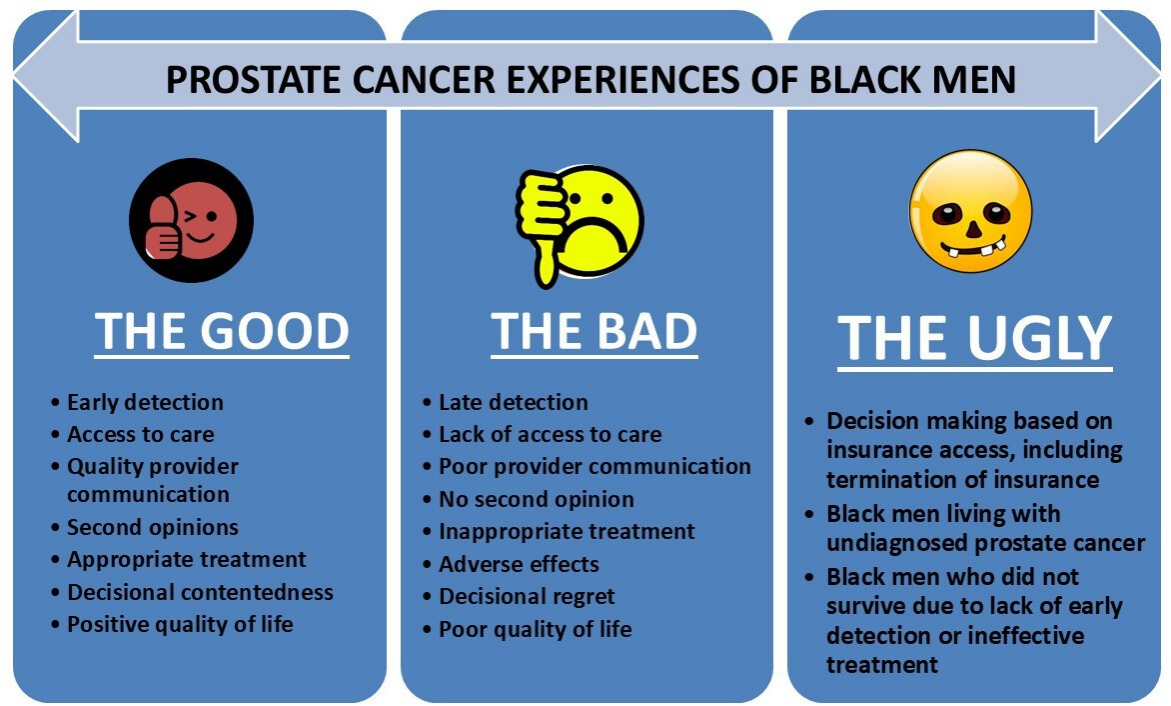
Prostate cancer experiences of Black men.

The CaPCaS model (Figure 1) and a CaP survivor-led CaP Experiences of Black men (Figure 2), developed by our research team [22-24], provided guidance on the individual-level factors to target for the CoLLab study. These include factors impacting CaP prevention, screening, diagnosis, treatment, survivorship, and advocacy, in addition to cross-cutting and contextual factors.

#### Intervention Planning Framework

The proposed intervention for this study was also guided by the Intervention Planning Framework [25,26]. The Intervention Planning Framework is a 5-step systematic approach used to design, implement, and evaluate health interventions, including (1) the proximal logic model of change that clearly states performance objectives, changeable determinants, and outcomes; (2) theoretical methods and practical strategies; (3) program design, which operationalizes the strategies into plans, designing of materials, pretest materials, and production of the materials; (4) an adoption and implementation plan; and (5) a monitoring and evaluation plan. It emphasizes integrating evidence, theory, and contextual factors to ensure interventions are effective, feasible, and culturally appropriate. Our academic-community team completed the first 2 steps prior to this study. The logic model of change that was developed is provided in Textbox 1, summarizing the program inputs, outputs, and impact. The theoretical methods and practical strategies are summarized in Table 1.

Textbox 1. **Community living lab logic model of change.**

Inputs (What we invest)

- Study team and resources; American Legion Posts team and resources; Institutional resources; Department of Defense Funding; Community Living Lab (CoLLab) Staff: community health workers (CHWs) and prostate cancer (CaP) Advocates; Collaborating organizations; CoLLab intervention manual; Time; Equipment; Space; Technology; Training; Other resources.

Outputs

Reach (Who we touch):

- Black men who are veterans and Black men (general public).

Activities (What we do):

- CaP care and survivorship (CaPCaS) activities across the cancer care continuum provided in the community, including education, training, consulting, navigating, clinical trials matching, dissemination, screening, webinars, social support services, digital health access, publications, presentations, evaluation.

Products and Process (What we develop/process):

- Products include Community Living Labs, educational materials, newsletters, and community reports.
- Process indicators include acceptability, fidelity, applicability, and contextual factors.

Outcomes/Impact

Short-term (Knowledge and learning):

- Improve Black men’s CaPCaS (Goal 1): awareness, knowledge, attitudes, health beliefs, perceived control, self-efficacy, skills, habits, intentions, and motivations.
- Increase Black men’s clinical trials (Goal 2): awareness, knowledge, attitudes, perceived control, intentions, and motivations.

Intermediate (Utilization and change in action):

- Improve Black men’s CaPCaS experiences and behavior (Goal 3): healthier lifestyle (diet, exercise), informed CaP screening, better CaP diagnosis experiences, treatment shared decision making, treatment adherence, symptom relief, minimal treatment side effects, involvement in advocacy, joining a research team.
- Increase Black men’s volunteering for, enrolling in, and participation in clinical trials (Goal 4).

Long-term (Impact and public good):

- Advance health equity and reduce disparities in prostate cancer (Goal 5).
- Improve quality of life, overall health, and wellness for individuals impacted by prostate cancer (Goal 6).

**Table 1.** Community Living Lab learning health system: theoretical methods and practical strategies.

Theoretical methods	Practical strategies
Goal	Create innovation environments in minoritized communities to address CaP^a^ disparities across the cancer care continuum and improve clinical trials diversity.
Target population	Black men.
Stakeholders	Community clinicians, patients, residents, and veterans.
Role of users	Share ideas, planning, provide training, codevelopment, provide feedback, educate, and evaluation.
Program components	Education, navigation, and support services focused on (1) social determinants of health; (2) prostate cancer risk reduction, prevention, screening, diagnosis, treatment, survivorship, and advocacy; and (3) cancer clinical trials. A special emphasis will be placed on cancer prevention, based on the American Association for Cancer Research (AACR) White Paper on Future of Cancer Prevention and targeting the prevention behaviors outlined in the model [27].
Program scope	(1) Identify the needs of Black men; (2) meet the needs through education, navigation, and support services; (3) evaluate services and programs for continuous improvement; and (4) document the reach and impact of services and programs.
Theory and evidence-based change methods	The following models guide the interventions: CaPCaS^b^ model [28-30] and Intervention Planning Framework [7,8].
Delivery applications for intervention [31]	
Communication sources	Physician/clinicians | prostate cancer survivors | advocates | researchers.
Communication content	Prostate Cancer Care Continuum | quality of life | etiology | statistics | charts | latest developments | research participation or clinical trials | reasons for health disparities | lifestyle factors/behavioral changes | distinguish between slow growing and fast growing cancer | aggressive versus non aggressive tumors | risk factors for Black men | treatment decision-making | patient navigation | studies with immediate implication | genetic studies | how to determine if a study is scientifically relevant so participants can also learn how to interpret insults.
Approach	Survivor stories | Use of humor | | Headline message | Simple charts, using percentages in lay format | Tailored information, eg, to age and educational level | Add FAQs^c^ | Call to action |
Communication product	Digital platforms at sites | website | | podcast | social media, especially Facebook and X (formerly Twitter) | video | print
Distribution channels	Smartphone/cell phone | social media, especially Facebook and X (formerly Twitter) | emails| radio (eg, Christian) | TV | Black media | Back of a football card – sporting event | car bumper sticker | work in collaboration with other outreach programs | prostate organizations that provide information to patients and survivors
Location	American Legion Posts in Duval and St. Johns Counties, Florida.
Promotion sites	Churches | Health Summit | primary care settings | recreational centers | sporting events | fraternal organizations | barber shops | family reunions | physician offices | community health centers

a CaP: prostate cancer.

b CaPCaS: CaP care and survivorship.

c FAQ: frequently asked question.

### Study Overview

This study is conducted by members of the iCCaRE Consortium, established in 2022 to address the disproportionate burden of CaP among Black men worldwide through research, research training, community engagement, and outreach (iccareconsortium.org). Four ALPs in Black communities in Florida were selected, with 3 serving as intervention sites (ALPs 197, 244, and 194) and one as a comparative site (ALP 187). The study focuses on the final 3 steps of the Intervention Planning Framework:

- Step 3: Program Design – Translating strategies into operational plans, including materials development, design, pretesting, and production.
- Step 4: Adoption and Implementation Plan – Establishing procedures and structures to support intervention delivery.
- Step 5: Monitoring and Evaluation Plan – Developing processes for ongoing assessment of implementation fidelity, outcomes, and impact.

The study design is a 30-month, mixed methods, pragmatic clinical trial to evaluate the feasibility, effectiveness, and scalability of this community-based intervention.

### Research Intervention and Comparator Sites

The research setting is ALPs in Northeast Florida. The Posts selected for this IDEA award are highly active in their communities, located in underserved minority neighborhoods, and primarily have minority veteran members. The CoLLab sites included one Hub in Duval County (Post 197) and 2 Spokes, Posts 244 in Duval County and 194 in St. Johns County. The comparative site was Post 184 in Delan County.

### Community Involvement in Program Design

**Figure 3.**
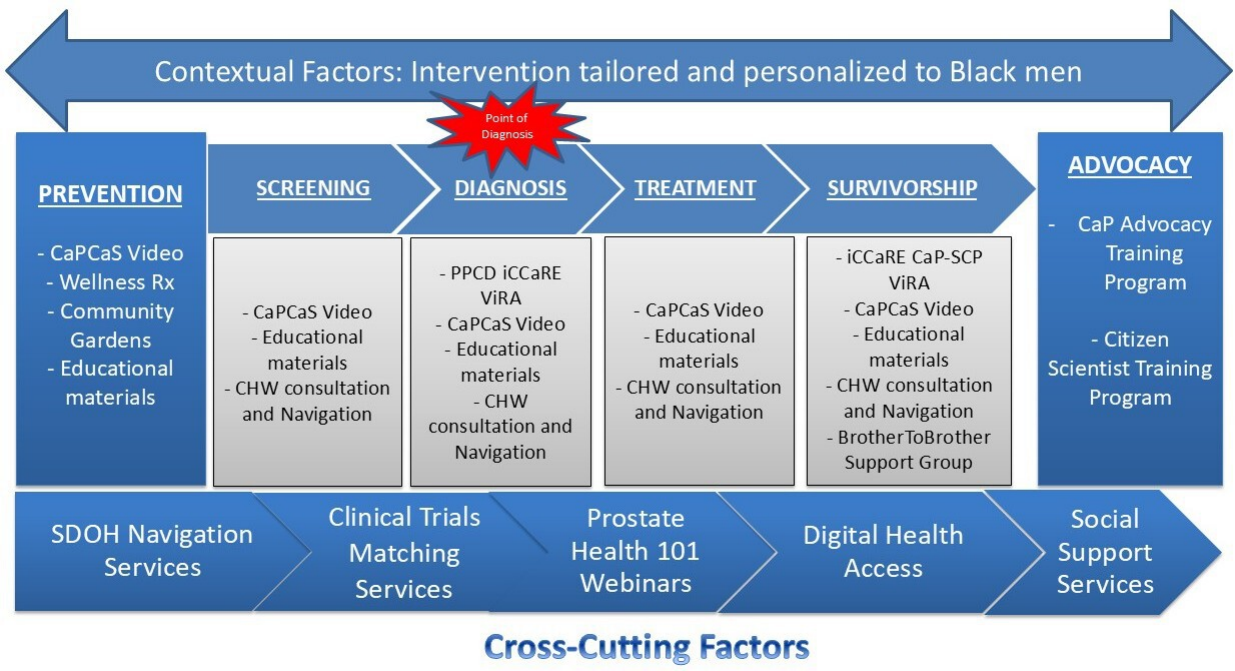
Community living lab intervention framework. CaPCaS: CaP care and survivorship; CHW: community health worker; SDOH: social determinants of health; ViRA: Virtual Robot Assistant.

The CoLLab program’s prostate health REACH services were co-designed through collaboration with community advisory boards (CABs) at each Post, each composed of 7 community members who recommend, review, and adjust services based on local needs. Data to guide CAB decisions were collected via focus groups, interviews, stakeholder consultations, and existing cancer needs assessments. CAB recommendations were then translated into site-specific REACH program designs by a multidisciplinary design team, including study staff, Learning Health System managers, and iCCaRE service representatives. Figure 3 summarizes the adapted CoLLab Intervention Framework.

**Figure 4.**
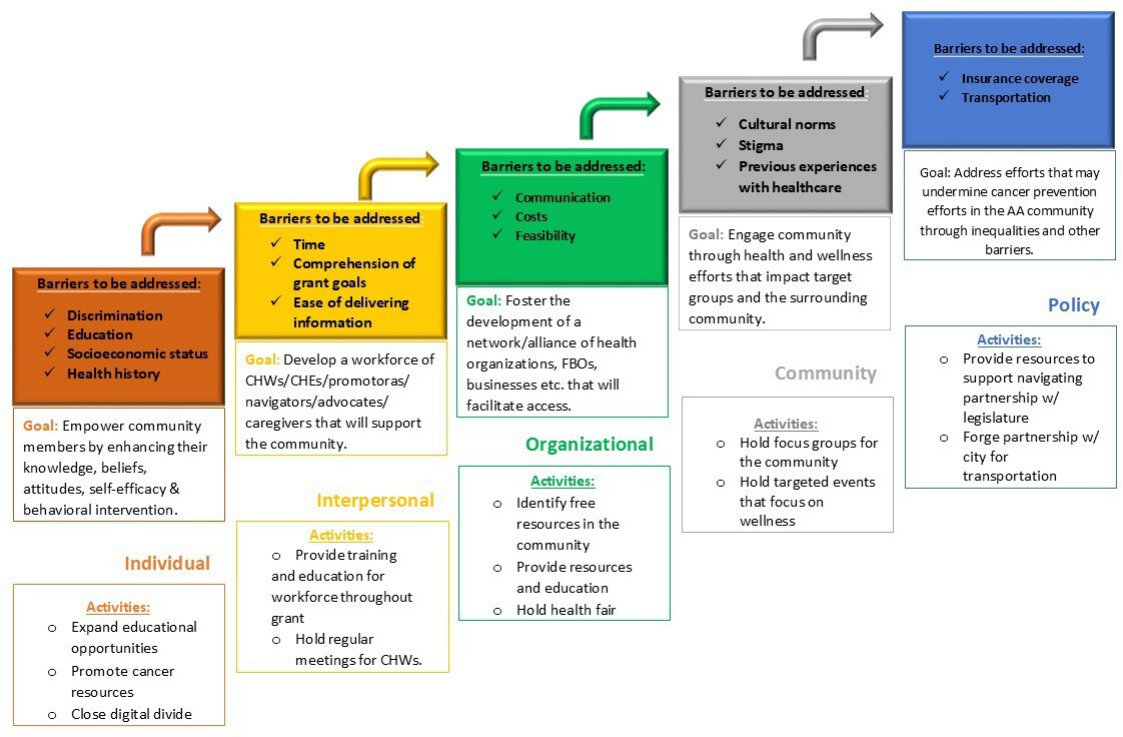
Community living lab social ecological model. CHW: community health worker.

During the program design phase, community members emphasized the importance of targeting interpersonal, community, and policy-level factors, in addition to individual-level determinants, to improve overall community health. This resulted in us adopting another model for the study, the social ecological model [32]. The social ecological model is a theoretical framework that emphasizes the multiple levels of influence on individual behavior, including intrapersonal (individual), interpersonal (relationships), organizational, community, and policy/societal levels. It recognizes that health outcomes are shaped not only by individual factors but also by other factors such as social networks, institutional contexts, community environments, and broader societal structures. This emphasizes the need for multi-level interventions to promote health and prevent disease. With input from community stakeholders, Figure 4 outlines the barriers to be addressed for the REACH services, the objectives of the intervention, and the proposed activities for the CoLLab Services.

The CoLLab Services integrated the following infrastructure, resources, and materials that have been proven effective in other programs.

- The SDOH Navigation Services is modeled after the American Academy of Family Physicians’ EveryONE Neighborhood Navigator, providing navigation for community members for food, housing, transportation, employment aid, legal aid, and financial support within or near the zip code.
- The Mayo Clinic Wellness RX program was developed in 2016 to address the Jacksonville Community’s health needs assessment. The wellness program includes: (1) wellness events that have covered topics that include mental health, cancer awareness, financial health, and heart disease; (2) provision of educational materials; (3) interactive demonstrations, such as cooking and exercising; and (4) grocery distribution to address food deserts. The Wellness Rx program will be offered annually at each of the intervention Post sites.
- The Mayo Clinic Florida Mobile Research Unit brings Mayo Clinic research resources/information into urban and rural hard-to-reach communities. It supports health disparities/community-based research studies (including clinical trials) to improve knowledge and access to clinical research.
- The WORD on Prostate Cancer was developed by our team [33] and is available on YouTube. It is an educational video, with the setting in a barbershop, and educates Black men about CaP screening.
- The CaPCaS educational videos [34] were developed by our team and comprise several educational videos based on the experiences of ethnically-diverse Black men (native-born and foreign-born) on CaP prevention, screening, detection, treatment, survivorship, and advocacy; all found to be efficacious in educating Black men and supporting their CaPCaS journey.
- The Mayo Clinic Community Research Registry was developed by our team in 2020. It includes proactively enrolling individuals in a research registry database, which is subsequently used to match them to Mayo Clinic clinical trials and biomedical research. This IRB-approved unique database includes demographic information, participant chronic disease data, and individual level of interest in research studies (minimal risk, specimen collection, etc) in order to tailor matches of actively recruiting research studies to minority participants.
- The iCCaRE Virtual Robot Assistant (ViRA) was developed in 2023 to support Black men from the point of CaP diagnosis through survivorship. ViRA [35] is a computer-generated holographic display of a human Artificial Intelligence therapist accessible through smartphones and AR glasses/headset. Black men with CaP often find it difficult to make informed decisions about their care, either due to fear, past experiences, perceived risks, lack of inclusion in the decision-making process, or just the complex nature of the decisions. The iCCaRE ViRA is personalized to Black men to help them make more informed decisions about their care and survivorship based on their data and personal preferences. Using real-time data and assisted by virtual/augmented reality technology, ViRA empowers patients and provides critical care from the diagnosis and beyond.
- The Citizen Scientist Training program focuses on developing the workforce for research advocacy to ensure that research is tailored to the priorities of underserved populations. Based on the National Institute of Health/National Institute of Environmental Health Sciences Community-Engaged Research and Citizen Science model, the program goal is to inform, educate, and empower health advocates and CHWs as research advocates.
- The CaP Advocacy training program trains cancer survivors and advocates to engage their communities, develop, and implement cancer health and survivorship programs by: (1) mobilizing the resources within their communities for health promotion, prevention, and survivorship strategies; (2) partnering with community key stakeholders; (3) raising funds to support advocacy activities; and (4) developing and organizing community-centered programs.

### Participants' Eligibility and Data Collection

The target study population is Black men between the ages of 30 and 80 years in Northeast FL. Being a community engagement research project, the target populations will be fully engaged at each stage of the research implementation. Thus, there is likely to be a modification to the proposed strategy based on input from the working group and CAB at each site. The prostate health REACH services at each site will be promoted through active and continuous community engagement and outreach led by CHWs. Black men residing near the intervention ALPs will be targeted to access the services.

### Participants Recruitment

**Figure 5.**
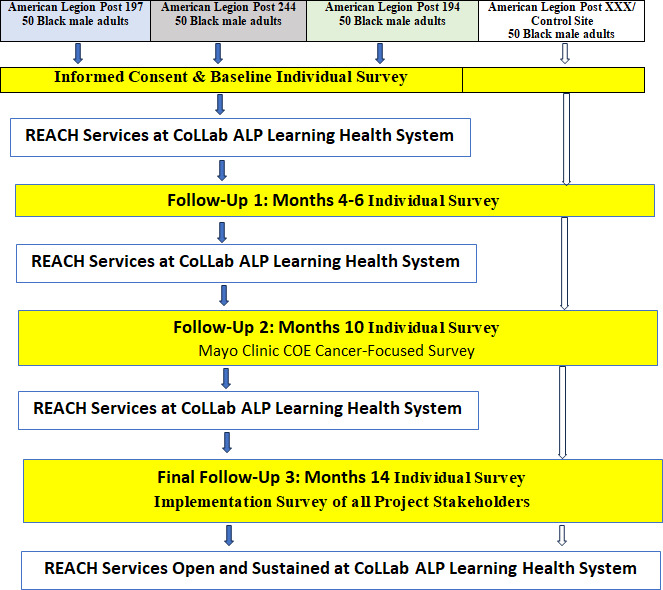
Community living lab study research design. CoLLab: Community Living Lab; REACH: Resources, Education, Amenities, and Community Health.

Figure 5 provides a summary of the data collection steps. For the adoption and program implementation, our goal is to recruit a total of 150 early adopters and users for the program (50 per site) and 50 participants at the comparison Post. Participants are actively recruited by CHWs safely through door-to-door visits, introducing the CoLLab REACH services, or dropping off information about the CoLLab Health System. We also partnered with local media to introduce the CoLLab Health System. Each site had a Grand Opening date, with community members invited to the Grand Opening. The participants will be followed over a minimum of one year.

### Study Timelines

The participants at intervention sites will be asked to visit their respective CoLLab Health System site at least twice a month and up to 24 times over one year to use any of the services. Additionally, the CoLLab sites will be open to the public. Data will be collected in-person or by phone 4 times over one year, at baseline and every 4 months (months 4, 8, and 12).

### Data Collection, Assessment, and Evaluation Plan

The CoLLab Health System sites will operate similarly to any support service center. A CHW will be on site to welcome Black men and provide an overview of the services that are available. S/he will then provide an intake form for the participant to state the kind of services they would like to access. The intake form will ask for first name, race, ethnicity, birth year, and residential zip code. Additionally, participants will check services that they would like from a list. Subsequently, the services will be provided or made available to the participant. Regardless of the service (even use of a computer to access cancer information), participants will be required to complete the intake form to capture use. Data will be collected in-person or by phone 4 times over one year, at baseline and every 4 months. At each stage, the data collection steps are as follows:

1. Informed consent will be secured by the interviewer prior to data collection.
2. The survey data will be collected using REDCaP, a web-based, clinical data-capture tool available for any research project at Mayo Clinic.
3. Pretest survey will be administered at enrollment for both intervention and comparative group participants.
4. Follow-up survey will be conducted every 4 months, with data collected a total of 4 times.
5. A US $20 incentive will be provided to all participants for each survey administration.

### Individual Assessment

The individual assessment will survey 200 participants, with 150 recruited at the intervention Posts and 50 recruited at the comparison Post. Using a pretest-posttest design, we will evaluate the impact of the CoLLab prostate health REACH services. In addition to evaluating the impact, we will also use the data and care experiences to improve the services continuously. As this is an exploratory short-term intervention, we will assess short-term goals of knowledge/learning improvement focused on the goals to (1) improve Black men’s CaP awareness, knowledge, attitude, health beliefs, perceived control, intentions, and cues to action; and (2) increase Black men’s clinical trials’ awareness, knowledge, attitude, perceived control, intentions, and cues to action. Using the questionnaire adapted from the validated CaPTC/AC3 Standardized Global Behavioral and Epidemiological Measures for Prostate Cancer Studies in Black Men [36,37]*,* we will collect data at baseline, month 3, 6, 9 and assess changes in (1) CaP-related knowledge, attitude, health beliefs, perceived control, intentions, and cues to action; and (2) clinical trials-related knowledge, attitude, perceived control, intentions, and cues to action. In addition, we will assess how many of the men sign up for the Mayo Clinic Community Research Registry, an indication of their interest in being matched to clinical trials at Mayo Clinic, as well as their participation in clinical trials. The study variables are described below.

- Knowledge of CaP is participants’ understanding of CaP information, including prevention, risk reduction, screening, detection, treatment, survivorship, and advocacy. Responses will be recorded as True, False, or Don’t Know, with each correct answer coded as 1, and incorrect or “Don’t Know” responses coded as 0. The total score ranged from 0 to 5, representing the number of correct knowledge items. Scores will be categorized as Poor Understanding (1-2), Moderate Understanding (3), and Full Understanding (4-5).
- Attitude is a positive or negative evaluation of CaP. It will be measured with 3 items with a 5-point Likert scale ranging from Very Unfavorable (1) to Very Favorable (5). Scores will be summed to create a composite score ranging from 3 to 15, where higher values denote more positive screening attitudes. Based on the scores, participants will be categorized as having a favorable attitude (score range of 12‐15), neutral (score range of 7‐11).
- Health belief questions will assess perceived susceptibility*,* which is the opinion of the chances of getting CaP; perceived severity, which is the opinion about the seriousness of CaP; and perceived behavioral control*,* which is the confidence of participants’ ability to screen for CaP. These will be assessed through 6 questions with Likert-type items with responses ranging from Strongly Disagree (1) to Strongly Agree (5). The sixth item (“Making a decision about screening is difficult”) will be reverse-coded. The overall belief score will range from 6 to 30, and will be categorized as strong (14-30), neutral (13‐23), or weak (6-12).
- CaP cues to action are exposure to information likely to activate CaP risk-reduction, prevention, and informed screening behaviors. These will be operationalized as engagement with 6 health prompts (eg, receiving a doctor’s recommendation or exposure to prostate cancer information). Each “Yes” response will be coded 1, and “No” responses will be 0, resulting in a total score from 0 to 6. Participants with a score range of 5‐6 will be categorized as having high cues to action, 3‐4 as neutral, and 1‐2 as low.
- Knowledge of CaP Clinical Trials is participants’ understanding of the CaP clinical trials information. The measures will follow the same rubric for the knowledge of CaP, with higher scores indicating higher knowledge about clinical trials.
- Attitude refers to positive or negative evaluations of clinical trials.
- Clinical Trial Participation Request, Intention, and Behavior focus on participants’ intention to participate in clinical trials within the next year, assess if they were asked to participate in a CaP clinical trial within the last year, and if they participated in a CaP clinical trial within the last year. The measure will be scored with Likert-type items with responses ranging from Strongly Disagree (1) to Strongly Agree (5), with a higher score attributed to a higher likelihood of clinical trial participation.
- Clinical trials cues to action are exposure to information likely to activate clinical trials participation behaviors. The questionnaire item will be a “Yes” response and “No” response.

Participants in the intervention group will also be asked to provide their satisfaction with and perceived quality of the CoLLab prostate health REACH services. The demographic characteristics of all participants and information on access to medical services will also be assessed.

### Statistical Analyses

Responses obtained from data collection will be analyzed using a PC-SAS program. The internal consistency of the study scales will be calculated to estimate their reliability. For each scale, item-remainder correlation will be assessed for item analysis. To assess the impact of the prostate health REACH services at CoLLab Learning Health System sites on study outcomes, the collected data will be evaluated for data quality and then summarized and analyzed. The data will be summarized using standard descriptive statistics for demographic and outcome variables. At baseline, comparisons of the early adopter group and the comparative group on study variables will be done to assess the similarity of the groups using appropriate tests, such as the *t* test, Wilcoxon test, or chi-square test, and 95% CIs. Generalized linear mixed regression approaches, such as repeated analysis of covariance, logistic mixed-effects regression, or a linear mixed-effects model, based on the types of outcomes, will be considered to study the differences between the study variables for the intervention and control groups. At baseline and months 4, 8, and 12, a direct comparison between the early adopter group and the comparison group will be carried out. In addition, trends in outcome variables for each of the 2 groups will be examined to compare the incremental differences in the outcome variables among the Black men over one year. We anticipate seeing a significant improvement in the study outcomes for the early adopter group at months 4, 8, and 12. Over the one-year intervention period, the trend analyses should show significant differences between both groups.

### Monitoring and Evaluation

#### Community Assessment

The community-level assessment data will be collected through the annual Mayo Clinic Comprehensive Cancer Center Community Cancer-focused Needs Assessment. The Mayo Clinic conducts an annual needs assessment that captures the following variables in the Cancer Center catchment areas, which include both study counties: demographics, health status, health care access, information seeking, and health information access, engagement with Cancer Center, cancer history (personal and family), health behaviors, community attributes, mental health/wellbeing, and clinical trials participation. Participants within the study counties will be purposefully oversampled during the study period. Focusing on the following variables, we will compare the study counties controlling for demographic variables: health status, health care access, information seeking and health information access, cancer history, health behaviors, and clinical trials participation. First, the data will be summarized using standard descriptive statistics for demographic and outcome variables. Subsequently, comparisons of the intervention county (Duval and St. Johns) and the comparative county (Volusia) on study variables will be conducted to assess the similarity of the groups using appropriate tests, such as the *t* test, Wilcoxon test, or chi-square test, and 95% CIs.

#### Additional Program Assessment

Additional program evaluation will include (1) assessing the reach of the CoLLab Health System prostate health REACH services*,* including extent and patterns of use, number of people reached over the period of the intervention, and demographics of people reached; and (2) the number of people who were successfully navigated for SDOH, clinical trials, and health care. The information from both individual-level and community-level assessments will be used to refine the services.

#### Assessment of Program Replication

To assess the feasibility of replicating the CoLLab Learning Health System and its services in Black communities nationally, we will estimate the direct and indirect costs of transplanting the CoLLab Learning Health System at ALPs in marginalized and minoritized communities. Micro-costing (detailed inventory, measurement, and valuation of each separate cost item) will be used to document the following: (1) start-up costs for development, including personnel, equipment, and training program costs; and (2) costs to implement the services. Based on the actual dollars spent from the grant, we will measure direct costs as follows: identifying all resources used and placing a dollar value on the resources (associated costs). The indirect cost associated with the CoLLab Learning Health System will be the cost of any institutional (Mayo Clinic and ALP) funding and resources used to support the program.

We will also explore the feasibility of transplanting the CoLLab Learning Health System and its services in Black communities nationally through formative interviews and documentation of costs associated with the program. Our ultimate goal is to successfully replicate the CoLLab Learning Health System and its prostate health REACH services. Establishing the process for replication is an exploratory aim and will provide us with feedback on the optimal approach for its implementation in Black communities nationwide. Using formative interviews of the experiences of key personnel and users, investigators will work with the program CAB, CHWs, and early adopters to address the following issues: “Why did the program work, or not work?” “Should program changes or improvements be made to increase the likelihood of success or to address changes in the services or problem situations?” “Were lessons learned that could help make future programs more successful?” and “What other ways can the services be presented? Are there other ways that the services can be promoted and progress tracked?*”* The responses obtained will be developed into a preliminary report: *“*Implementation of the CoLLab Learning Health System in Black Communities.”

### Ethical Considerations

This study will be conducted according to the Declaration of Helsinki. This study received ethical approval from the Mayo Clinic Institutional Review Board (IRB ID: 23‐007988) on August 20, 2024. It was subsequently reviewed by the US Army Medical Research and Development Command (USAMRDC), Office of Human and Animal Research Oversight (OHARO), and Office of Human Research Oversight (OHRO). On September 25, 2024, the USAMRDC OHARO OHRO approved it as a minimal risk study for the enrollment of a maximum of 250 participants. All eligible participants will provide written informed consent prior to being enrolled in this pragmatic trial, and all participants will receive an incentive according to the provisions in the IRB-approved protocol. Only deidentified data of participants will be used for analysis and evaluation to ensure the confidentiality of participants’ information.

## Results

### Engagement With Community Advisory Board

The study commenced after IRB approval, with the nomination of 7 CAB members at each site, with a total of 21 CAB members. The CABs at each intervention site recommended the prostate health REACH services adopted.

### Interventions and Site Activation

Multiple REACH services and programs were available to be adopted at each site based on the needs of Black men. Examples include the following:

1. The iCCaRE Consortium SDOH Navigation Services, which was modeled after the American Academy of Family Physicians EveryONE Neighborhood Navigator, provides navigation for community members for food, housing, transportation, employment aid, legal aid, and financial support within or near the zip code.
2. The Wellness RX program was implemented to address some health needs of the communities around the posts. The wellness program includes wellness events, interactive demonstrations, such as cooking and exercising, and grocery distribution to address food deserts.
3. The Prostate Health Support group is a monthly support group led by CaP survivors and interested participants within the community. Issues around prostate health are discussed with emphasis on the need for screening, early diagnosis, and the pathway to survivorship.
4. The Educational resources and videos on Prostate Cancer [38] are educational videos, with the setting in a barbershop, that educate Black men on CaP prevention, screening, detection, treatment, survivorship, and advocacy, and other resources to be shared with the participants.
5. The CaP Advocacy training program was also developed to train cancer survivors and advocates to engage their communities, develop, and implement cancer health and survivorship programs.

### Training of CHWs for Delivery of Intervention

Before the activation of the above intervention, CHWs were trained to be able to deliver CoLLab prostate health REACH services and navigation from January to March 2024. Their involvement is essential to the success of the CoLLab Learning Health System. Two phases of paid training were developed to prepare CHWs for their roles.

The first phase, General Training, was facilitated by a certified CHW trainer from one of our community partner organizations. Using an approved curriculum from the Florida CHW Certification Board, she provided 30 hours of instruction and supported trainees in preparing for the CHW certification examination. Following completion of the required curriculum, she continued to offer updates and continuing education in alignment with Florida CHW certification requirements.

The second phase, Prostate Health REACH Services Training, was delivered by members of the research team and included both didactic instruction and supervised practice in April 2024. Study team members engaged in role-play as early adopters to support skill development. A nongraded quiz followed each didactic module to assess comprehension, and results were shared with trainees to promptly address any areas of confusion. CHWs also conducted a 30-minute “mock intervention” with a team member. These sessions were audio- and video-recorded for review by the training team, who used the recordings to provide feedback and strengthen CHW competencies.

The third phase of CHW capacity building includes continuous training and retraining scheduled throughout the study period. The first was the CHW retreat held in January 2025, and the second was held in January 2026. We anticipate holding another retreat by September 2026.

### Data Collection

Data collection commenced in October 2024. Baseline recruitment for the intervention arm has been completed, with 195 Black men enrolled across intervention ALPs. To date, 11 participants have been enrolled at the control ALP, and recruitment for the control arm remains ongoing. Once data analyses are completed, the results will be shared scientifically through scientific conferences and publications. In addition, community reports will be developed and disseminated publicly.

## Discussion

### Principal Findings

The CoLLab study addresses a longstanding and well-documented public health challenge of the disproportionate burden of CaP among Black men as well as the persistent structural, interpersonal, behavioral, and systemic barriers that shape their cancer care experiences [3]. Despite decades of research, Black men continue to face inequities across the CaP continuum, driven by multilevel determinants spanning health-system factors, provider-level dynamics, and individual-level constraints. The CoLLab Learning Health System model represents an innovative, community-driven strategy to intervene at multiple levels simultaneously by integrating real-time learning, cocreation, trusted CHWs, and culturally grounded REACH services into community environments where Black men already gather.

The use of a precision community health framework strengthens the contextual relevance of this intervention. Precision community health approaches emphasize designing interventions based on localized cultural, behavioral, environmental, and social contexts rather than population-level generalizations [6,28]. By grounding CoLLab activities in community-derived data—through CAB input, focus groups, interviews, and existing needs assessments—this study ensures that intervention components align with the lived experiences and priorities of Black men in Northeast Florida. This approach increases cultural congruence, fosters sustained engagement, and supports the development of long-term community infrastructures for prostate health promotion.

A major strength of the CoLLab model is its integration of CHWs as frontline navigators and educators, a strategy supported by extensive evidence showing that CHWs improve cancer knowledge, navigation, and outcomes among underserved populations [7-9]. Their trusted role within communities enhances communication effectiveness, increases service uptake, and mitigates historical mistrust of health systems. The 2-phase training program equips CHWs with both general competencies and prostate-specific content, ensuring they can deliver tailored education and support across the CaP continuum. Their involvement also aligns with core CoLLab Learning Health System features, including continuous learning, rapid feedback integration, and real-time quality improvement [6].

The digital divide poses an additional challenge to achieving cancer care equity for Black men. Prior research shows that although they use mobile devices at high rates, gaps in broadband access, digital literacy, and trust in digital health tools persist and contribute to inequity in cancer screening, communication, and clinical trial participation [15-17]. By embedding digital tools—including internet access points, evidence-based educational media, and culturally adapted technologies such as ViRA—within community-based CoLLab sites, this study provides a mechanism to introduce digital health resources in a supportive environment. This strategy may help reduce technology-related barriers documented in the literature, including usability challenges, unfamiliarity with digital interfaces, and cultural perceptions that digital interactions lack credibility compared with face-to-face communication [16-18].

The design of this study enhances real-world relevance and supports exploration of both implementation feasibility and preliminary impact. Mixed methods evaluation, integration of a comparison site, and systematic tracking of service utilization allow for a nuanced understanding of how CoLLab functions within community settings. This approach is consistent with emerging literature on community-engaged, context-adapted cancer control interventions, which highlight the importance of iterative feedback, cocreation, and flexible implementation strategies in achieving population-level impact [10,11].

The CoLLab study also lays essential groundwork for future replication and scalability. Micro-costing methods and formative interviews with CHWs, CAB members, and early adopters will yield practical insights into what is required to transplant this model into other Black communities. Literature on Living Labs and community-centered health innovation emphasizes the importance of understanding implementation context, resource demands, and user-experience data to support broad dissemination [4,5,28]. Findings from this study will help inform future implementation science research evaluating real-world effectiveness and long-term sustainability.

### Limitations

Several limitations warrant consideration. First, as a feasibility trial conducted under controlled conditions, this study is designed to assess efficacy rather than real-world effectiveness; future implementation science studies will be needed to evaluate scalability and sustainability [29]. Participant attrition presents another risk, as differential dropout could compromise group equivalence; however, analytic strategies controlling for baseline characteristics will mitigate this threat. Repeated survey administration may also introduce testing effects, though evidence suggests that spacing assessments reduces this bias [27,30]. Finally, as a community engagement research initiative, some CoLLab components may evolve based on CAB feedback, introducing variability across sites. Such adaptations are consistent with community-engaged research frameworks but may complicate comparisons across intervention sites.

Despite these limitations, this protocol addresses important evidence gaps in CaP inequities research by testing a comprehensive, community-driven model that integrates multilevel determinants—individual, interpersonal, community, and structural. The CoLLab Learning Health System advances current approaches by combining CHW-based navigation, tailored education, digital access, and continuous learning into a scalable framework capable of supporting sustained prostate health equity. If successful, CoLLab may serve as a national model for implementing community-based Learning Health Systems that enhance CaP prevention, early detection, survivorship, and clinical trial participation in marginalized communities.

We recognize that the success of our efforts to address CaP burden depends not only on rigorous research implementation but also on the effective dissemination of findings to both scientific and, critically, lay communities. To ensure broad and meaningful reach, we will implement a structured dissemination strategy coordinated through the iCCaRE Consortium. This strategy will include the publication and public release of community reports summarizing key initiatives and findings; presentations at community health events; and targeted press releases through local Black media outlets.

In addition, we will leverage the iCCaRE Consortium YouTube Channel [39] and related digital platforms designed to translate scientific discoveries for the public, moderated by a scientist and a CaP survivor. These platforms—including a dedicated virtual reality environment, YouTube channel, podcast, and integrated social media distribution—will be used to share updates and outcomes from the CoLLab Learning Health System directly with Black communities. We will actively monitor the reach, engagement, and impact of these dissemination activities to ensure that research findings are accessible, culturally relevant, and responsive to community information needs.

### Conclusions

Building a healthier community requires strategies that originate from within those communities—strategies grounded in an understanding of barriers to healthy behavior, inclusive principles of community engagement, and authentic, trust-building approaches [31]. Using a precision community health framework, we applied granular, community-specific data to design interventions tailored to the unique context of the CoLLab Learning Health System. Leveraging the substantial foundation established through the iCCaRE Consortium and the Mayo Clinic Comprehensive Cancer Center, this study will establish the feasibility of developing a CoLLab Learning Health System in Black communities with the overarching goal of improving access to prostate health resources, education, amenities, and community supports for Black men.

By delivering immediate access to prostate health REACH services in underserved communities, the study aims to influence the full CaP care continuum—from prevention to survivorship—and enhance participation in CaP clinical trials. Specifically, the CoLLab Learning Health System seeks to increase CaP knowledge and awareness; shape and reinforce perceptions, beliefs, and attitudes; encourage informed and proactive health behaviors; and dispel myths and misconceptions. It will further support Black men in making informed decisions regarding participation in CaP clinical trials. Over the long term, this model will foster sustained behavior change and help users navigate and overcome systemic barriers associated with CaP. Collectively, these contributions position the CoLLab Learning Health System as a meaningful step toward advancing prostate health equity in underserved communities.

## Supplementary material

10.2196/89330 Peer Review Report 1 Peer-review report from the US Army Medical Research and Development Command Congressionally Directed Medical Research Programs, Prostate Cancer Research Program Idea Development Award, Detection, Diagnosis and Prognosis Review Panel (Department of Defense, United States).

## Abbreviation

ALP: American Legion Post
CAB: community advisory board
CaP: prostate cancer
CaPCaS: CaP care and survivorship
CHW: community health worker
CoLLab: Community Living Lab
OHARO: Office of Human and Animal Research Oversight
OHRO: Office of Human Research Oversight
REACH: Resources, Education, Amenities, and Community Health
SDOH: social determinants of health
USAMRDC: US Army Medical Research and Development Command
ViRA: Virtual Robot Assistant
WM: White men
