# Supplementary material for: Feasibility of Establishing Community Living Labs (CoLLabs) to Improve Access to Prostate Health Resources, Education, Amenities, and Community Health: Protocol for a Pragmatic Clinical Trial
Source: JMIR Res Protoc. 2026 May 15;15:e89330. doi: 10.2196/89330 (PMC13179051; doi:10.2196/89330)
Supplement: Peer Review Report 1 [file resprot-v15-e89330-s001.pdf]

Department of Defense
U.S. Army Medical Research and Development Command
Congressionally Directed Medical Research Programs
Fiscal Year 2022 Prostate Cancer Research Program
Idea Development Award - Established Investigator
Peer Review Summary Statement

CDMRP Log Number: PC220312
Grants.gov ID Number: GRANT13684179
Review Panel: Detection, Diagnosis and Prognosis
Discussion Period: 09/22/2022-09/23/2022

Project Duration: 30 months
Budget Requested: $1,190,974
Direct Costs: $900,000
Indirect Costs: $290,974

Title: Feasibility of Establishing Community Living Labs to Improve Access to Prostate Health Resources, Education, Amenities, and Community Health (REACH) for Black Men
Principal Investigator: Folakemi Odedina
Performing Organization: Mayo Clinic and Foundation, Jacksonville
Contracting Organization: Mayo Clinic and Foundation, Jacksonville

OVERVIEW

The Principal Investigator (PI) of this application hypothesizes that improving social determinants of health (SDOH) factors and prostate cancer care and survivorship (CaPCaS)-related factors and fostering participation in clinical trials will lead to an improvement in outcomes related to prostate health. The project's specific aims are (1) to cocreate real-life user-centric Community Living Lab (CoLLab) Learning Health System at 3 American Legion posts, with Post 197 as the hub and 2 other posts in Duval County, Florida, as spokes; (2) using a pretest-posttest design, to evaluate the impact of the CoLLab Learning Health System; and (3) to explore the feasibility of replicating the CoLLab Learning Health System in Black communities nationally, starting with inclusive Cancer Care Research Equity (iCCaRE) institutions and organizations.

	Average Score	Standard Deviation
**Overall Evaluation**<br>*Rating Scale: 1.0 (highest merit) to 5.0 (lowest merit)*	1.8<br>**(Excellent)**	0.1
**Criteria**<br>*Rating Scale: 10 (highest merit) to 1 (lowest merit)*	**Average Score**	
**Innovation**	9.0	
**Impact**	8.5	
**Research Strategy and Feasibility**	6.1	
**Personnel**	8.4	

Procurement Sensitive Document
Do not copy or distribute without CDMRP written permission.
Prepared by SRA International, Inc., A GDIT Company, a support contractor to CDMRP

SCORED CRITERIA

Innovation
Average Score: 9.0

Scientist Reviewer A

Strengths: This study is innovative in its concept and focus. The idea of a community living lab is not new, but it is novel to consider it in context of prostate health services. The setting will be American Legion posts, which is also a novel idea for a partnership targeting Black men (civilians and those with military history) for prostate health resources. Community living labs have not been used previously to address prostate cancer disparities. Additionally, this living lab will be developed as a health system, providing education and navigation services to prospective patients.

The application outlines a novel conceptual model, developed by the research team, that will be used to define the framework for the living lab. The Prostate Cancer Care and Survivorship model will guide the individual-level intervention services that will be provided from prevention/screening to survivorship. Unlike many other studies, this approach will cater to all community members, not just one segment of the community at one point on the prostate cancer journey.

Weaknesses: No weaknesses were noted.

Scientist Reviewer B

Strengths: The proposed CoLLab Learning Health System is a novel approach that has never been used to study and address prostate cancer disparities. This is the first time that a living lab has been proposed to address prostate cancer in Black men with the intervention residential in the Black community. There is no published study that has reported the creation of community learning health systems to address prostate cancer disparities in Black men.

Weaknesses: No weaknesses were noted.

Impact
Average Score: 8.5

Scientist Reviewer A

Strengths: The FY22 PCRP overarching challenges that will be addressed in this application are advancing health equity and reducing disparities in prostate cancer as well as improving quality of life to enhance outcomes and overall health and wellness for those impacted by prostate cancer. If successful, this application will build upon an iterative process of implementation science projects by these investigators with these community partners. This study will lay the foundation for implementation studies and additional studies of individual and community-level outcomes across the cancer care continuum utilizing the established community living labs which will be strategically placed to target Black men, a high-risk demographic for poor prostate cancer outcomes.

The PI also plans to evaluate and tweak interventions as necessary to optimize the outcomes that will lead to a decrease in prostate cancer disparities across the patient to survivorship journey. The strategic placement of community sites at the American Legion posts, where there has been an established partnership with the PI, to target Black and military members/veterans and their families is a primary reason that this study can be impactful.

Procurement Sensitive Document
Do not copy or distribute without CDMRP written permission.

Weaknesses: No weaknesses were noted.

Scientist Reviewer B

Strengths: The application addresses 2 of the FY22 PCRP overarching challenges: advancing health equity and reducing disparities in prostate cancer and improving quality of life to enhance outcomes and overall health and wellness for those impacted by prostate cancer. The strengths include both short-term and long-term impact. In the short term, the proposal will provide information and navigation services on multiple factors that affect prostate cancer disparities, which can be adopted for public health interventions. The long-term expected outcomes for the CoLLab Learning Health System outlined in the application are also recognized.

Weaknesses: The selection of 200 Black men to evaluate the impact of the program may not be representative of a larger population.

Consumer Reviewer

Strengths: The application states that it addresses the following FY22 PCRP overarching challenges: improving quality of life to enhance outcomes and overall health and wellness for those impacted by prostate cancer and advancing health equity and reducing disparities in prostate cancer. The application is focused on developing a community-based health system called Community Living Lab (COL LAB). The COL LAB learning health system provides an innovative community setting that provides health-related education in highly accessible community sites. One of the long-term goals of the application is to eliminate prostate cancer morbidity and mortality disparities for Black men through community-based and culturally appropriate interventions.

Weaknesses: The application discusses expanding the health centers to other locations after being successful at the initial location but does not clearly discuss how the effectiveness or success will be measured.

Discussion Notes

Panel members recognized a high potential for impact within the prostate cancer community. However, during discussion, questions were raised regarding declining American Legion membership and how this may diminish the impact of this application.

Research Strategy and Feasibility
Average Score: 6.1

Scientist Reviewer A

The application is an exploratory feasibility study focused on making prostate cancer care and survivorship services and clinical trials accessible to Black men. A mixed-methods approach, specifically a descriptive analysis of pre-post tests/surveys, and discussions during town hall meetings will be used to guide the intervention and evaluation across sites. The locations of the 3 American Legion posts in Duval County, Florida, were selected based on county demographics. Compared to the state percentages and rates, these counties had a relatively large percentage of Black residents, higher poverty rates, and higher prostate cancer mortality rates for both Black and White men. The posts selected for this study are highly active (providing various resources and programs) in their underserved minority communities. For this study, Black men ages 30-80 will be recruited, and the target populations will be fully engaged in each stage of the study methodology.

For Aim 1, the implementation of the program will occur. The Resources, Education, Amenities, and Community Health (REACH) services and programs will be administered by research staff and trained community health workers (CHWs). An intervention framework based on previous work by the consortium that the partnership is embedded in will provide the model for the intervention design.

For Aim 2, the impact of the intervention will be evaluated with pre- and post-surveys of 200 participants: 150 from the early adopter Duval County posts and 50 from a comparison post in St Johns County, Florida. The post-surveys will be collected at 4, 8, and 12 months for direct comparisons between the Duval County posts and the St Johns County post. The surveys will obtain data on participants' prostate cancer knowledge, attitude, perceived severity, perceived behavioral control, cues to action, and knowledge of prostate cancer clinical trials, clinical trials perceived behavioral control, and clinical trial participation request, intention, behavior, and cues to action. Community-level comparisons will be conducted via the annual Mayo Clinic Cancer Center Community Cancer-Focused Needs Assessment Survey at the county level. Descriptive analyses and generalized linear mixed regression models will be used to study differences between the intervention and control groups. The assessments will also include the reach of the living lab (number of people reached and demographics) and the number of people who were successfully navigated to resources for social determinants of health, clinical trials, and health care.

For Aim 3, the cost of transplanting the community living labs to other American Legion posts in marginalized and minoritized communities will be assessed using information generated from the cost of the current project and with formative interviews from current program workers about what worked and how what did not work can be improved.

Strengths: The application's rationale is that a comprehensive program that meets the many needs of Black men across the prostate cancer care continuum will help reduce prostate cancer disparities and improve the health and well-being of prostate cancer patients and their families. The Community Living Lab provides this comprehensive resource. The reviewed literature and preliminary data provide a strong foundation for the continuation of this partnership and development of the living lab. The hypothesis, aims, and methods are appropriate for this exploratory study.

During the development and testing of the living lab, potential problems and alternative approaches are addressed appropriately with protocol and statistical adjustments and future study design plans. An appropriate statistical plan is included. Plans for obtaining needed resources and utilizing existing resources are also included.

Multiple approaches will be used to educate participants, communicate with community members, distribute learning lab products, and promote the program such as the use of social media, various community and clinical settings, radio, TV, community news, and bumper stickers, which will help with broad distribution of information.

Validation of measurement tools has been completed. Pretesting of the REACH services will be conducted by CHWs with previously validated measures before implementation at the posts. Every aspect of the protocol has been carefully considered and planned. There is also institutional buy-in from Mayo Clinic, which helps ensure the sustainability of the program.

Weaknesses: It's unclear if the REACH services will be administered only virtually by program staff or if there will also be in-person sessions. More details are needed on how CHWs will be assigned and if an onsite presence during specific hours is required or by appointment only to access the materials and products that will be made available at the Community Living Labs. A diagram/flowchart of the study protocol/methods would be useful, as there are many moving parts at multiple sites.

Procurement Sensitive Document
Do not copy or distribute without CDMRP written permission.

It is also unclear if all 200 participants will be required to explore all of the resources available or if they will self-select the resources. Self-selection, which is a more personalized approach, may be more of a realistic approach but also may limit the number of observations that can be analyzed. Finally, the recruitment plan does not detail how the men will be recruited at each site or how many men typically attend current prostate cancer events at the posts.

Scientist Reviewer B

Strengths: Strong preliminary data are provided regarding prostate cancer care and survivorship among Black men and developed, or developing, culturally responsive interventions across the cancer care continuum.

Weaknesses: To evaluate the impact of the application, 200 Black men will be selected. However, there is insufficient information on how those individuals will be selected except for their age range and location. It is important to identify individuals that are representative of a larger population. For example, it is important to ensure that there are individuals with different demographics, educational backgrounds, some with family history of prostate cancer, and different risk factors. Given such heterogeneity among individuals, a sample size of 200 is viewed as undersized. There is also insufficient information regarding the nature of the survey, how the survey questions would be determined, and how the data will be analyzed.

Discussion Notes

Several shortcomings were discussed by panel members, and these notably diminished enthusiasm for this application. Among the topics discussed were the view that the application provides insufficient information concerning the patient population to be studied and concerns regarding the survey instruments to be used. It was recognized that these surveys will be used to judge study effectiveness, but it was unclear to the panel members how they will be analyzed.

Personnel
Average Score: 8.4

Scientist Reviewer A

Dr Folakemi Odedina is the PI of this study. The PI received a PhD in pharmaceutical sciences with a focus on health behavior and outcomes in 1994 from the University of Florida. The PI is currently a professor of hematology/oncology, the deputy director of the Mayo Clinic Comprehensive Cancer Center, the associate director of the Center for Health Equity and Community Engagement Research, and the director of the Global Health Equity Program at Mayo Clinic in Jacksonville, Florida.

Strengths: The PI is an internationally renowned prostate cancer disparities researcher. As the associate director of Community Outreach and Engagement, she and her multidisciplinary team along with their long-time community partners are well positioned to establish Community Living Labs in targeted, high-burden areas in Florida and elsewhere. Key areas for successful intervention are covered by the team (health disparities, community engagement, clinical services, behavioral health, and biostatistics consulting, CHWs, and trusted staff of the American Legion posts).

Mr Arnold Merriweather will serve as a Coinvestigator and will colead the project with Dr Odedina. He is a 12-year prostate cancer survivor and prostate cancer advocate who has led programming at the primary American Legion post selected in Duval County. Dr Trevanne Matthews Hew is a study Coinvestigator. Dr Hew is a practicing physician and serves as a senior associate consultant in the Division of

Hematology/Oncology at Mayo Clinic. Dr Jennifer Crook is a Coinvestigator and is currently a postdoctoral trainee in health equity and community engagement at Mayo Clinic. Ms Monica Albertie is a Coinvestigator. She serves as the director of research operations for the Office of Community Outreach and Engagement for the Mayo Clinic Comprehensive Cancer Center.

Weaknesses: A letter from Mr Merriweather detailing his continued commitment and role would be helpful to understanding the tasks that he will lead and how much time will be given to each task. Also, it is unclear if the level of onsite effort will be sufficient. This concern is based on how CHWs will be assigned.

Scientist Reviewer B

Strengths: The PI has directed over 40 research projects, mostly using community-based participatory approaches, mixed-methods approaches, and translational behavioral research principles. The PI was awarded by the American Society of Health-Systems Pharmacy and the Association of Black Health-System Pharmacists with the Inaugural Leadership Award in health disparities. Her efforts in training underrepresented minorities was recognized through the INSIGHT Into Diversity 2016 Inspiring Women in STEM Award.

This application utilizes an outstanding team of researchers. The PI has assembled a strong team including investigators with expertise in biostatistics, clinical health psychology, public health, business and health administration, and hematology/oncology.

Weaknesses: No weaknesses were noted.

UNSCORED CRITERIA

Budget

Scientist Reviewer A

The budget appears to be appropriate for the proposed research.

Scientist Reviewer B

The budget is appropriate for the proposed project. The budget is well justified with respect to salaries and wages of senior and other personnel.

Environment

Scientist Reviewer A

The intellectual property plan is appropriate. The scientific environment is optimal for this project occurring as a partnership between community sites and the Mayo Clinic Comprehensive Cancer Center. Mayo Clinic provides resources for health equity and community engagement, clinical resources, and a biostatistics and informatics core, all of which are essential for this research project. There are letters of support from academic and community collaborators. There are institutional letters of support for facilities and resources that will be required for this work.

Procurement Sensitive Document
Do not copy or distribute without CDMRP written permission.

Scientist Reviewer B

The scientific environment is appropriate for the proposed research.

Data and Research Resource Sharing Plan

Scientist Reviewer A

There is a clear plan to make these research products and data available to outside investigators. There is also a hope to expand these efforts beyond the Mayo Clinic catchment area to community sites around the United States, potentially with other collaborators.

Scientist Reviewer B

The data and research resources sharing plan is appropriate for the proposed project.

Application Presentation

Scientist Reviewer A

Overall, the application is clear and well written. There are a few spelling/grammar errors in the application. Flowcharts could help summarize some of the text to make it easier to follow the multifaceted protocol.

Scientist Reviewer B

The application is well presented. Clear writing and thought give confidence in the clear logic and future implementation of proposed work. The graphics are easy to see with correct scaling and clarity.

Procurement Sensitive Document
Do not copy or distribute without CDMRP written permission.
